# (1*S*,8*R*,15*S*,19*R*)-17-Benzyl-17-aza­penta­cyclo­[6.6.5.0^2,7^.0^9,14^.0^15,19^]nona­deca-2(7),3,5,9(14),10,12-hexa­ene chloro­form monosolvate

**DOI:** 10.1107/S1600536812037749

**Published:** 2012-09-08

**Authors:** Ielyzaveta Bratko, Sonia Ladeira, Nathalie Saffon, Emmanuelle Teuma, Montserrat Gómez

**Affiliations:** a Laboratoire Hétérochimie Fondamentale et Appliquée, UMR CNRS 5069, Université Paul Sabatier, 118 route de Narbonne, 31062 Toulouse Cedex 9, France; b Université de Toulouse, UPS, Institut de Chimie de Toulouse FR2599, 118 route de Narbonne, 31062 Toulouse Cedex 9, France

## Abstract

In the title compound, C_25_H_23_N·CHCl_3_, the dihydro­anthracene unit is bent with a dihedral angle between the benzene rings of 57.82 (8)°. The N atom of the pyrrolidine heterocycle, which has an envelope conformation with the N atom as the flap, exhibits a pronounced pyramidalization [Σ(C—N—C) = 328.07°], indicating an accentuated N-donor character. In the crystal, this behaviour is evident by the C—H⋯N hydrogen bond involving a solvent mol­ecule and the N atom. The absolute configuration at the C-atom fused positions of the pyrrolidine group were crystallographically confirmed to be *S* and *R*.

## Related literature

For catalytic applications of 9,10-dihydro­anthracene-succinimides and their related pyrrolidine derivatives, see: Sasaoka *et al.* (2006 ▶); Sanhes *et al.* (2009 ▶, 2010 ▶). For the synthesis of these ligands, see: Sanhes *et al.* (2008 ▶). For a description of the Cambridge Structural Database, see: Allen (2002 ▶).
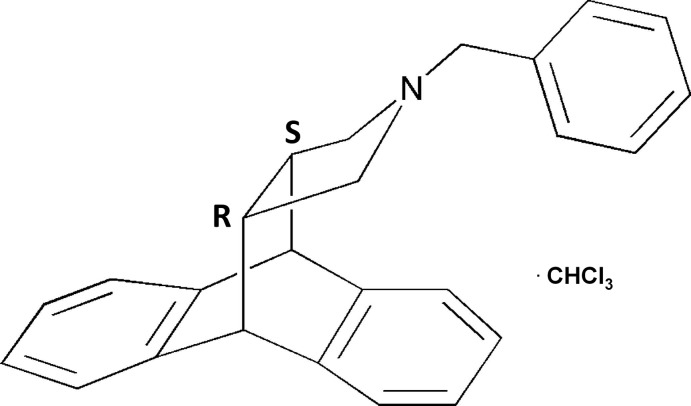


## Experimental

### 

#### Crystal data

C_25_H_23_N·CHCl_3_
*M* *_r_* = 456.81
Monoclinic, 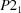

*a* = 8.6455 (2) Å
*b* = 10.7338 (3) Å
*c* = 12.3310 (3) Å
β = 99.055 (1)°
*V* = 1130.04 (5) Å^3^
*Z* = 2
Mo *K*α radiation
μ = 0.42 mm^−1^
*T* = 193 K
0.80 × 0.70 × 0.40 mm

#### Data collection

Bruker SMART APEXII diffractometer
Absorption correction: multi-scan (*SADABS*; Bruker, 2006 ▶) *T*_min_ = 0.730, *T*_max_ = 0.850
19688 measured reflections
6678 independent reflections
6147 reflections with *I* > 2σ(*I*)
*R*_int_ = 0.021

#### Refinement

*R*[*F* ^2^ > 2σ(*F* ^2^)] = 0.042
*wR*(*F* ^2^) = 0.119
*S* = 1.04
6678 reflections
271 parameters
1 restraint
H-atom parameters constrained
Δρ_max_ = 0.59 e Å^−3^
Δρ_min_ = −0.54 e Å^−3^
Absolute structure: Flack (1983 ▶), 3057 Friedel pairs
Flack parameter: −0.01 (5)

### 

Data collection: *APEX2* (Bruker, 2006 ▶); cell refinement: *APEX2* and *SAINT* (Bruker, 2006 ▶); data reduction: *SAINT*; program(s) used to solve structure: *SHELXS97* (Sheldrick, 2008 ▶); program(s) used to refine structure: *SHELXL97* (Sheldrick, 2008 ▶); molecular graphics: *ORTEP-3 for Windows* (Farrugia, 1997 ▶); software used to prepare material for publication: *SHELXTL* (Sheldrick, 2008 ▶) and *publCIF* (Westrip, 2010 ▶).

## Supplementary Material

Crystal structure: contains datablock(s) global, I. DOI: 10.1107/S1600536812037749/su2490sup1.cif

Structure factors: contains datablock(s) I. DOI: 10.1107/S1600536812037749/su2490Isup2.hkl

Additional supplementary materials:  crystallographic information; 3D view; checkCIF report

## Figures and Tables

**Table 1:** Hydrogen-bond geometry (Å, °)

*D*—H⋯*A*	*D*—H	H⋯*A*	*D*⋯*A*	*D*—H⋯*A*
C26—H26⋯N1	1.00	2.36	3.320 (2)	161

**Fig. 1.**
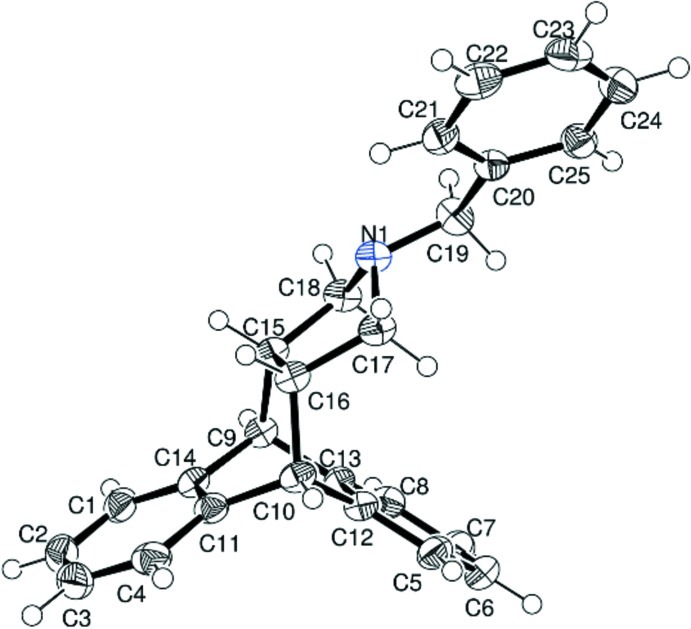
The molecular structure of the title compound with the atom numbering. The displacement ellipsoids are drawn at the 50% probability level. The solvent molecule has been omitted for clarity.

**Fig. 2.**
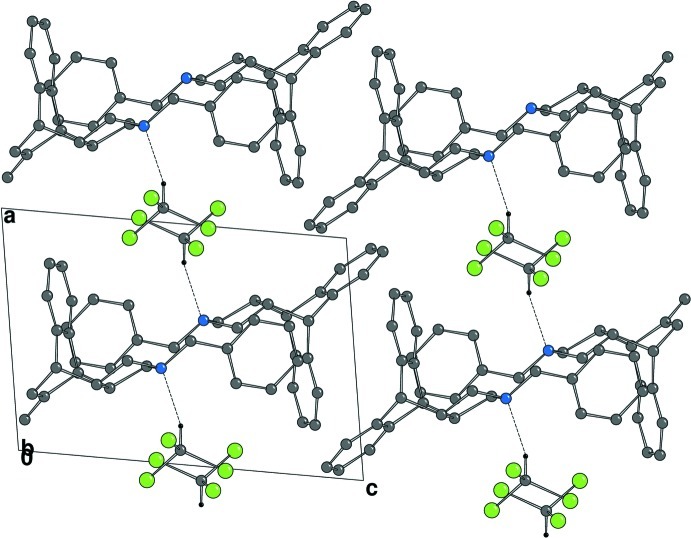
A view along the b axis of the crystal packing of the title compound, showing the C—H···N hydrogen bond (dashed line) involving the solvent molecule. H atoms not involved in hydrogen bonding have been omitted for clarity.

## supplementary crystallographic information

### Comment

9,10-Dihydroanthracene-succinimides are target molecules for pharmaceutical and medical uses (Sanhes *et al.*, 2008), and their related pyrrolidines have also found applications as organocatalysts (Sasaoka *et al.*, 2006; Sanhes *et al.*, 2009; 2010). The synthesis of these compounds is mainly based on thermal-promoted Diels-Alder cycloadditions (for the succinimide derivatives), followed by chemical reduction to give the corresponding heterocyclic amines [Sanhes *et al.*, 2008]. From a structural point of view, a large number of 9,10-dihydroanthracene-succinimides have been analyzed by X-ray single-crystal diffraction. A search of the Cambridge Structural Database gave 67 hits (CSD, version 5.33, update No. 4, August 2012; Allen, 2002), however, no crystallographic data is available for the corresponding non-substituted pyrrolidine ligand. Herein, we report on the synthesis and crystal structure of the title compound.

The molecular structure of the title compound is shown in Fig. 1. The 9,10-dihydroanthracenyl is bent with a dihedral angle between the benzene rings of 57.82 (8)°. The pyrrolidine heterocycle has an envelope conformation with atom N1 as the flap. It is displaced from the mean plane of the four C-atoms, C15—C18 [maximum deviation = 0.0025 (15) Å] by 0.6313 (14) Å. This mean plane forms a dihedral angle of 50.78 (10)° with the C20—C25 benzyl ring. In contrast to analogous dicarboximide compounds, a pronounced pyramidalization of the atom N1 is observed with Σ C—N1—C = 328.07°, which signifies an accentuated N-donor character.

In the crystal, this N-donor behaviour is evident by the C—H···N intermolecular hydrogen bond involving a chloroform solvate molecule (Table 1 and Fig. 2).

The absolute configuration of atoms C15 and C16 was crystallographically confirmed to be *S* and *R*, respectively (Fig. 1).

### Experimental

The title compound was prepared by the reduction of the corresponding 9,10-dihydroanthracene-succinimide following the reported procedure (Sanhes *et al.*, 2008). To a solution of the succinimide (920 mg, 2.52 mmol) in 50 ml of THF at 273 K, LiAlH_4_ (1.43 g, 37.7 mmol) was added in small portions and the mixture was then refluxed for 72 h. The reaction mixture was cooled to 273 K, then diethylether (30 ml) and an aqueous saturated solution of Na_2_SO_4_ were sequentially added. The precipitate that formed was filtered off and the filtrate washed three times with water. The combined organic layers were dried over anhydrous Na_2_SO_4_, filtered and the solvent evaporated under vacuum. Crystals of the title compound were obtained by slow evaporation of a solution in CHCl_3_. The title compound was characterized by high-resolution mass spectrometry (CI, dichloromethane): calc. mass 338.19; found: 338.19 (for C_25_H_23_N). Further spectroscopic data for the title compound is available in the archived CIF.

### Refinement

All the H atoms were included in calculated positions and treated as riding atoms: C—H = 0.95 Å (aromatic), 0.99 Å (methylene) and 1.00 Å (methine,) with *U*_iso_(H) = 1.2*U*_eq_(C).

### Crystal data

**Table:**

C_25_H_23_N·CHCl_3_	*F*(000) = 476
*M**_r_* = 456.81	*D*_x_ = 1.343 Mg m^−^^3^
Monoclinic, *P*2_1_	Mo *K*α radiation, λ = 0.71073 Å
Hall symbol: P 2yb	Cell parameters from 9897 reflections
*a* = 8.6455 (2) Å	θ = 2.4–34.7°
*b* = 10.7338 (3) Å	µ = 0.42 mm^−^^1^
*c* = 12.3310 (3) Å	*T* = 193 K
β = 99.055 (1)°	Block, colourless
*V* = 1130.04 (5) Å^3^	0.80 × 0.70 × 0.40 mm
*Z* = 2	

### Data collection

**Table:**

Bruker SMART APEXII diffractometer	6678 independent reflections
Radiation source: fine-focus sealed tube	6147 reflections with *I* > 2σ(*I*)
Graphite monochromator	*R*_int_ = 0.021
phi and ω scans	θ_max_ = 30.5°, θ_min_ = 2.4°
Absorption correction: multi-scan (*SADABS*; Bruker, 2006)	*h* = −12→12
*T*_min_ = 0.730, *T*_max_ = 0.850	*k* = −15→15
19688 measured reflections	*l* = −17→17

### Refinement

**Table:**

Refinement on *F*^2^	Secondary atom site location: difference Fourier map
Least-squares matrix: full	Hydrogen site location: inferred from neighbouring sites
*R*[*F*^2^ > 2σ(*F*^2^)] = 0.042	H-atom parameters constrained
*wR*(*F*^2^) = 0.119	*w* = 1/[σ^2^(*F*_o_^2^) + (0.0672*P*)^2^ + 0.2655*P*] where *P* = (*F*_o_^2^ + 2*F*_c_^2^)/3
*S* = 1.04	(Δ/σ)_max_ = 0.001
6678 reflections	Δρ_max_ = 0.59 e Å^−^^3^
271 parameters	Δρ_min_ = −0.54 e Å^−^^3^
1 restraint	Absolute structure: Flack (1983), 3057 Friedel pairs
Primary atom site location: structure-invariant direct methods	Flack parameter: −0.01 (5)

### Special details

**Table:**

Experimental. Spectroscopic data for the title compound: ^1^H NMR (300 MHz in CDCl_3_): 7.31 – 7.34 (m, 7H, Harom), 7.24 – 7.27 (m, 2H, Harom), 7.14 – 7.19 (m, 4H, Harom), 4.20 (s, 2H, H9,10), 3.31 (s, 2H, H19), 2.87 (m, 2H, H17 or H18), 2.78 (m, 2H, H15,16), 1.90 (m, 2H, H17 or H18) ^13^C NMR (75 MHz in CDCl_3_): 143.9, 141.8 (C11,12,13,14), 128.8, 128.1, 125.8, 125.7, 125.6, 123.6 (CH arom), 60.0 (C19), 57.0 (C17,18), 47.3 (C9,10), 44.3 (C15,16).

### Fractional atomic coordinates and isotropic or equivalent isotropic displacement parameters (Å^2^)

**Table:**

	*x*	*y*	*z*	*U*_iso_*/*U*_eq_	
N1	0.39289 (16)	0.52587 (12)	0.43293 (11)	0.0266 (2)	
C1	0.1444 (2)	0.50798 (18)	−0.04276 (14)	0.0345 (3)	
H1	0.1492	0.5921	−0.0659	0.041*	
C2	0.0421 (2)	0.4244 (2)	−0.10382 (15)	0.0429 (4)	
H2	−0.0227	0.4516	−0.1689	0.051*	
C3	0.0345 (2)	0.3016 (2)	−0.06997 (17)	0.0435 (4)	
H3	−0.0364	0.2456	−0.1117	0.052*	
C4	0.1298 (2)	0.25967 (18)	0.02461 (15)	0.0352 (3)	
H4	0.1250	0.1754	0.0473	0.042*	
C5	0.6395 (2)	0.28010 (16)	0.18645 (14)	0.0326 (3)	
H5	0.6355	0.1963	0.2107	0.039*	
C6	0.7800 (2)	0.33116 (18)	0.16495 (15)	0.0376 (4)	
H6	0.8722	0.2816	0.1747	0.045*	
C7	0.7867 (2)	0.45386 (19)	0.12935 (15)	0.0365 (4)	
H7	0.8829	0.4871	0.1143	0.044*	
C8	0.65237 (19)	0.52818 (16)	0.11575 (13)	0.0309 (3)	
H8	0.6569	0.6122	0.0922	0.037*	
C9	0.35679 (17)	0.54536 (13)	0.12701 (12)	0.0255 (3)	
H9	0.3634	0.6325	0.0995	0.031*	
C10	0.34340 (18)	0.31443 (14)	0.19041 (13)	0.0265 (3)	
H10	0.3398	0.2246	0.2114	0.032*	
C11	0.23268 (18)	0.34320 (14)	0.08567 (13)	0.0280 (3)	
C12	0.50511 (18)	0.35394 (14)	0.17174 (12)	0.0260 (3)	
C13	0.51215 (17)	0.47792 (14)	0.13708 (12)	0.0253 (3)	
C14	0.23907 (18)	0.46747 (14)	0.05191 (12)	0.0268 (3)	
C15	0.30194 (18)	0.54093 (13)	0.24191 (12)	0.0250 (3)	
H15	0.1958	0.5797	0.2359	0.030*	
C16	0.29201 (17)	0.40197 (13)	0.27884 (12)	0.0253 (3)	
H16	0.1816	0.3822	0.2875	0.030*	
C17	0.39598 (19)	0.39743 (14)	0.39155 (13)	0.0285 (3)	
H17A	0.3534	0.3384	0.4410	0.034*	
H17B	0.5041	0.3720	0.3846	0.034*	
C18	0.4115 (2)	0.60275 (14)	0.33636 (12)	0.0293 (3)	
H18A	0.5213	0.6014	0.3226	0.035*	
H18B	0.3801	0.6901	0.3467	0.035*	
C19	0.5165 (2)	0.55150 (17)	0.52496 (13)	0.0337 (3)	
H19A	0.5195	0.6423	0.5391	0.040*	
H19B	0.6182	0.5276	0.5038	0.040*	
C20	0.49863 (19)	0.48467 (14)	0.63052 (12)	0.0272 (3)	
C21	0.3573 (2)	0.43277 (17)	0.64887 (14)	0.0325 (3)	
H21	0.2688	0.4345	0.5925	0.039*	
C22	0.3457 (2)	0.37825 (18)	0.75005 (16)	0.0387 (4)	
H22	0.2495	0.3418	0.7616	0.046*	
C23	0.4726 (3)	0.37670 (18)	0.83363 (14)	0.0390 (4)	
H23	0.4630	0.3412	0.9028	0.047*	
C24	0.6135 (2)	0.42704 (18)	0.81587 (14)	0.0385 (4)	
H24	0.7013	0.4259	0.8729	0.046*	
C25	0.6271 (2)	0.47953 (16)	0.71431 (14)	0.0325 (3)	
H25	0.7250	0.5122	0.7021	0.039*	
C26	0.0596 (2)	0.65871 (19)	0.47279 (19)	0.0438 (4)	
H26	0.1596	0.6107	0.4782	0.053*	
Cl1	−0.07721 (8)	0.59279 (8)	0.36846 (6)	0.06723 (19)	
Cl2	0.09629 (12)	0.81306 (8)	0.44019 (12)	0.1005 (3)	
Cl3	−0.00976 (9)	0.64798 (9)	0.59955 (6)	0.0731 (2)	

### Atomic displacement parameters (Å^2^)

**Table:**

	*U*^11^	*U*^22^	*U*^33^	*U*^12^	*U*^13^	*U*^23^
N1	0.0317 (6)	0.0213 (5)	0.0280 (6)	−0.0020 (4)	0.0080 (5)	0.0010 (4)
C1	0.0287 (7)	0.0446 (9)	0.0315 (7)	0.0070 (6)	0.0083 (6)	0.0030 (6)
C2	0.0287 (8)	0.0663 (13)	0.0335 (8)	0.0056 (8)	0.0043 (6)	−0.0026 (8)
C3	0.0276 (8)	0.0604 (12)	0.0430 (9)	−0.0087 (8)	0.0075 (7)	−0.0170 (9)
C4	0.0303 (7)	0.0368 (8)	0.0410 (8)	−0.0070 (6)	0.0141 (6)	−0.0106 (7)
C5	0.0333 (8)	0.0304 (7)	0.0349 (8)	0.0073 (6)	0.0079 (6)	−0.0003 (6)
C6	0.0291 (7)	0.0446 (10)	0.0398 (8)	0.0083 (7)	0.0077 (6)	−0.0056 (7)
C7	0.0257 (7)	0.0491 (10)	0.0363 (8)	−0.0037 (7)	0.0101 (6)	−0.0060 (7)
C8	0.0308 (7)	0.0327 (7)	0.0310 (7)	−0.0064 (6)	0.0108 (6)	−0.0013 (6)
C9	0.0279 (6)	0.0207 (6)	0.0290 (6)	−0.0007 (5)	0.0079 (5)	0.0030 (5)
C10	0.0295 (7)	0.0187 (5)	0.0331 (7)	−0.0007 (5)	0.0103 (5)	−0.0002 (5)
C11	0.0252 (6)	0.0293 (7)	0.0312 (7)	−0.0017 (5)	0.0096 (5)	−0.0039 (5)
C12	0.0279 (6)	0.0237 (6)	0.0276 (6)	0.0000 (5)	0.0083 (5)	−0.0017 (5)
C13	0.0251 (6)	0.0253 (6)	0.0262 (6)	−0.0002 (5)	0.0066 (5)	0.0003 (5)
C14	0.0246 (6)	0.0277 (6)	0.0297 (7)	0.0023 (5)	0.0088 (5)	−0.0001 (5)
C15	0.0291 (6)	0.0192 (5)	0.0280 (6)	0.0014 (5)	0.0091 (5)	0.0030 (5)
C16	0.0282 (6)	0.0202 (6)	0.0292 (6)	−0.0012 (5)	0.0096 (5)	0.0020 (5)
C17	0.0352 (7)	0.0224 (6)	0.0295 (7)	0.0027 (5)	0.0095 (6)	0.0034 (5)
C18	0.0388 (8)	0.0210 (6)	0.0292 (6)	−0.0031 (6)	0.0095 (6)	0.0013 (5)
C19	0.0354 (8)	0.0343 (8)	0.0316 (7)	−0.0085 (6)	0.0057 (6)	0.0019 (6)
C20	0.0295 (7)	0.0255 (6)	0.0271 (6)	0.0002 (5)	0.0063 (5)	−0.0032 (5)
C21	0.0289 (7)	0.0366 (8)	0.0330 (7)	0.0001 (6)	0.0084 (6)	0.0002 (6)
C22	0.0403 (9)	0.0402 (9)	0.0401 (9)	−0.0023 (7)	0.0201 (7)	−0.0001 (7)
C23	0.0529 (10)	0.0375 (9)	0.0296 (8)	0.0037 (7)	0.0155 (7)	−0.0003 (6)
C24	0.0427 (9)	0.0405 (9)	0.0310 (8)	0.0037 (7)	0.0014 (7)	−0.0054 (7)
C25	0.0309 (7)	0.0332 (7)	0.0332 (7)	−0.0026 (6)	0.0043 (6)	−0.0054 (6)
C26	0.0380 (9)	0.0372 (9)	0.0568 (11)	0.0008 (7)	0.0096 (8)	−0.0027 (8)
Cl1	0.0523 (3)	0.0910 (5)	0.0566 (3)	−0.0100 (3)	0.0033 (2)	−0.0085 (3)
Cl2	0.0901 (6)	0.0433 (3)	0.1741 (10)	−0.0087 (4)	0.0397 (6)	0.0149 (5)
Cl3	0.0745 (4)	0.0909 (5)	0.0582 (3)	−0.0120 (4)	0.0239 (3)	−0.0231 (3)

### Geometric parameters (Å, º)

**Table:**

N1—C19	1.457 (2)	C11—C14	1.401 (2)
N1—C17	1.4718 (19)	C12—C13	1.402 (2)
N1—C18	1.4782 (19)	C15—C18	1.532 (2)
C1—C14	1.386 (2)	C15—C16	1.5659 (19)
C1—C2	1.395 (3)	C15—H15	1.0000
C1—H1	0.9500	C16—C17	1.533 (2)
C2—C3	1.386 (3)	C16—H16	1.0000
C2—H2	0.9500	C17—H17A	0.9900
C3—C4	1.393 (3)	C17—H17B	0.9900
C3—H3	0.9500	C18—H18A	0.9900
C4—C11	1.397 (2)	C18—H18B	0.9900
C4—H4	0.9500	C19—C20	1.515 (2)
C5—C12	1.394 (2)	C19—H19A	0.9900
C5—C6	1.396 (3)	C19—H19B	0.9900
C5—H5	0.9500	C20—C21	1.393 (2)
C6—C7	1.392 (3)	C20—C25	1.394 (2)
C6—H6	0.9500	C21—C22	1.396 (2)
C7—C8	1.397 (3)	C21—H21	0.9500
C7—H7	0.9500	C22—C23	1.382 (3)
C8—C13	1.389 (2)	C22—H22	0.9500
C8—H8	0.9500	C23—C24	1.382 (3)
C9—C13	1.514 (2)	C23—H23	0.9500
C9—C14	1.515 (2)	C24—C25	1.395 (3)
C9—C15	1.564 (2)	C24—H24	0.9500
C9—H9	1.0000	C25—H25	0.9500
C10—C12	1.513 (2)	C26—Cl2	1.746 (2)
C10—C11	1.513 (2)	C26—Cl1	1.754 (2)
C10—C16	1.556 (2)	C26—Cl3	1.764 (2)
C10—H10	1.0000	C26—H26	1.0000
			
C19—N1—C17	113.31 (13)	C9—C15—C16	109.31 (11)
C19—N1—C18	111.26 (12)	C18—C15—H15	109.0
C17—N1—C18	103.50 (11)	C9—C15—H15	109.0
C14—C1—C2	119.54 (18)	C16—C15—H15	109.0
C14—C1—H1	120.2	C17—C16—C10	115.14 (12)
C2—C1—H1	120.2	C17—C16—C15	104.06 (12)
C3—C2—C1	120.33 (17)	C10—C16—C15	109.67 (11)
C3—C2—H2	119.8	C17—C16—H16	109.3
C1—C2—H2	119.8	C10—C16—H16	109.3
C2—C3—C4	120.64 (17)	C15—C16—H16	109.3
C2—C3—H3	119.7	N1—C17—C16	104.15 (12)
C4—C3—H3	119.7	N1—C17—H17A	110.9
C3—C4—C11	119.10 (18)	C16—C17—H17A	110.9
C3—C4—H4	120.4	N1—C17—H17B	110.9
C11—C4—H4	120.4	C16—C17—H17B	110.9
C12—C5—C6	118.98 (15)	H17A—C17—H17B	108.9
C12—C5—H5	120.5	N1—C18—C15	103.77 (12)
C6—C5—H5	120.5	N1—C18—H18A	111.0
C7—C6—C5	120.79 (16)	C15—C18—H18A	111.0
C7—C6—H6	119.6	N1—C18—H18B	111.0
C5—C6—H6	119.6	C15—C18—H18B	111.0
C6—C7—C8	120.20 (16)	H18A—C18—H18B	109.0
C6—C7—H7	119.9	N1—C19—C20	114.73 (13)
C8—C7—H7	119.9	N1—C19—H19A	108.6
C13—C8—C7	119.28 (16)	C20—C19—H19A	108.6
C13—C8—H8	120.4	N1—C19—H19B	108.6
C7—C8—H8	120.4	C20—C19—H19B	108.6
C13—C9—C14	106.76 (12)	H19A—C19—H19B	107.6
C13—C9—C15	107.63 (12)	C21—C20—C25	118.72 (15)
C14—C9—C15	105.46 (12)	C21—C20—C19	122.63 (14)
C13—C9—H9	112.2	C25—C20—C19	118.59 (14)
C14—C9—H9	112.2	C20—C21—C22	120.06 (16)
C15—C9—H9	112.2	C20—C21—H21	120.0
C12—C10—C11	106.76 (12)	C22—C21—H21	120.0
C12—C10—C16	108.06 (12)	C23—C22—C21	120.75 (17)
C11—C10—C16	105.24 (12)	C23—C22—H22	119.6
C12—C10—H10	112.1	C21—C22—H22	119.6
C11—C10—H10	112.1	C24—C23—C22	119.57 (16)
C16—C10—H10	112.1	C24—C23—H23	120.2
C4—C11—C14	120.17 (15)	C22—C23—H23	120.2
C4—C11—C10	126.34 (15)	C23—C24—C25	120.07 (16)
C14—C11—C10	113.49 (13)	C23—C24—H24	120.0
C5—C12—C13	120.26 (14)	C25—C24—H24	120.0
C5—C12—C10	126.29 (14)	C20—C25—C24	120.78 (16)
C13—C12—C10	113.45 (13)	C20—C25—H25	119.6
C8—C13—C12	120.49 (14)	C24—C25—H25	119.6
C8—C13—C9	126.03 (14)	Cl2—C26—Cl1	109.88 (13)
C12—C13—C9	113.48 (13)	Cl2—C26—Cl3	111.44 (12)
C1—C14—C11	120.22 (15)	Cl1—C26—Cl3	109.86 (12)
C1—C14—C9	126.33 (15)	Cl2—C26—H26	108.5
C11—C14—C9	113.45 (13)	Cl1—C26—H26	108.5
C18—C15—C9	115.86 (12)	Cl3—C26—H26	108.5
C18—C15—C16	104.31 (12)		

### Hydrogen-bond geometry (Å, º)

**Table:**

*D*—H···*A*	*D*—H	H···*A*	*D*···*A*	*D*—H···*A*
C26—H26···N1	1.00	2.36	3.320 (2)	161
